# Immunometabolic pathways in liver fibrosis: current evidence for selected plant-derived metabolites associated with traditional Chinese medicine

**DOI:** 10.3389/fphar.2026.1810588

**Published:** 2026-08-04

**Authors:** Xiaoxin Liu, Yanxi Li, Junke Guan, Lingfei Fan, Sitong Li, Waseem Hassan, Hammad Ahmed, Liyuan Cheng, Yingqi Wang

**Affiliations:** 1 Department of Pharmacy, School of Medicine and Health Sciences, Wuxi Taihu University, Wuxi, Jiangsu, China; 2 Department of English, School of Humanities and Law, Wuxi Taihu University, Wuxi, Jiangsu, China; 3 Department of Pharmacy, COMSATS University Islamabad, Lahore Campus, Lahore, Pakistan; 4 Department of Pharmacy, Sialkot Institute of Science and Technology, Sialkot, Punjab, Pakistan; 5 Department of Nursing, School of Medicine and Health Sciences, Wuxi Taihu University, Wuxi, Jiangsu, China

**Keywords:** antifibrotic, hepatic stellate cells, Kupffer cells, liver diseases, liver fibrosis, metabolic reprogramming, phytochemicals, traditional Chinese medicine

## Abstract

Liver fibrosis is a progressive consequence of chronic hepatic injury characterized by sustained activation of hepatic stellate cells (HSCs) within a complex network of metabolic and inflammatory signaling. This review critically examines selected plant-derived metabolites associated with herbs used in Traditional Chinese Medicine (TCM), including salvianolic acid B, curcumin, berberine, resveratrol, puerarin, glycyrrhizin, tetrandrine, oroxylin A, and schisandrin B, for their potential roles in modulating immunometabolic pathways relevant to fibrogenesis. A structured search of PubMed and Web of Science identified mechanistic, preclinical, and clinical studies. Overall, these compounds were reported to influence key pathways implicated in liver fibrogenesis, including glycolysis, mitochondrial dysfunction, lipid metabolism, NF-κB, TGF-β/Smad, PI3K–Akt–mTOR, and HIF-1α signaling. However, the strength of evidence varies considerably across compounds. Curcumin and resveratrol have the most consistent translational data, whereas salvianolic acid B, tetrandrine, and oroxylin A remain supported primarily by preclinical findings with limited mechanistic and clinical validation. Current evidence is constrained by model heterogeneity, reliance on associative pathway analyses, and the scarcity of histologically confirmed clinical outcomes. These findings suggest that selected phytochemicals associated with TCM herbs may modulate immunometabolic processes involved in liver fibrosis, but their therapeutic potential remains incompletely defined and requires confirmation in standardized, mechanistically informed clinical studies.

## Highlights

- Hepatic stellate cell (HSC) activation, driven by metabolic reprogramming rather than cytokines alone, is central to liver fibrogenesis.
- Key metabolic shifts in activated HSCs include enhanced aerobic glycolysis, altered mitochondrial respiration, increased glutaminolysis, and loss of lipid droplets.
- A perpetuating cycle exists where immune-fibrotic crosstalk (involving Kupffer cells, macrophages, and T-cells) reinforces both metabolic and pro-fibrotic signaling.
- Selected plant-derived metabolites isolated from botanical drugs used in Traditional Chinese Medicine modulate multiple immunometabolic pathways in experimental models and represent potential leads for future antifibrotic research.

## 1 Introduction

Liver fibrosis is a progressive consequence of chronic hepatic injury (Lyu et al., 2022; Schwabe et al., 2020; Pellicano et al., 2023; Roehlen et al., 2020; Böttcher and Pinzani, 2017) characterized by excessive extracellular matrix deposition and distortion of liver architecture (Stalnikowitz and Weissbrod, 2003; Guixé-Muntet et al., 2024; Somnay et al., 2024; Dhar et al., 2020). While early fibrosis may be reversible, sustained injury leads to cirrhosis and hepatocellular carcinoma. Central to this pathological process is the hepatic stellate cell (HSC), a perisinusoidal, lipid-storing cell that normally maintains sinusoidal integrity and participates in retinoid metabolism. Upon chronic injury, HSCs undergo a phenotypic conversion into proliferative, contractile myofibroblast-like cells that overproduce extracellular matrix (ECM) (Khomich et al., 2020), express α-smooth muscle actin (Kim et al., 2024) and secrete potent profibrotic mediators such as transforming growth factor-*β* (TGF-*β*) (Horn and Tacke, 2024).

The activation of HSCs, historically, was interpreted largely through transcriptional and cytokine-driven frameworks (De Smet et al., 2021). However, a decisive paradigm shift has emerged with the recognition that metabolic reprogramming is not merely a result of HSC activation but also a prerequisite and driver of the process (Roh et al., 2025). Recent evidence demonstrates that activated HSCs adopt a metabolic profile reminiscent of a Warburg-like phenotype in which increased glycolysis supplies both ATP and biosynthetic precursors necessary for ECM synthesis (Trivedi et al., 2021). The switch is characterized by a shift to aerobic glycolysis with upregulation of HK2, PFKFB3, and LDHA, generating biosynthetic intermediates and lactate that promotes fibrogenesis via GPR81 signaling (Khanal et al., 2024; Wang et al., 2019). Concurrently, oxidative stress-induced mitochondrial hyperactivity increases reactive oxygen species (ROS) generation, characterized by DRP1-mediated fission and elevated mtROS, activate p38 MAPK and NF-κB pathways, while impaired biogenesis exacerbates dysfunction (Zhou et al., 2022; Yang et al., 2022). Loss of retinoid-containing lipid droplets marks the metabolic transition from quiescence to activation, accompanied by disrupted fatty acid oxidation (FAO), impaired mitochondrial *β*-oxidation (Jophlin et al., 2018) and increased lipogenesis *via* CPT1A downregulation and SREBP-1c activation (Molenaar et al., 2017). Pathways such as mTORC1, HIF-1α, and PI3K–Akt become abnormally activated, reshaping glycolytic, oxidative and lipid metabolic fluxes. Enhanced glutaminolysis through GLS1, induced by TGF-*β* and hypoxia, supplies α-ketoglutarate to sustain the TCA cycle and collagen synthesis (Ge et al., 2018; Zhou et al., 2021).

**FIGURE 1:**
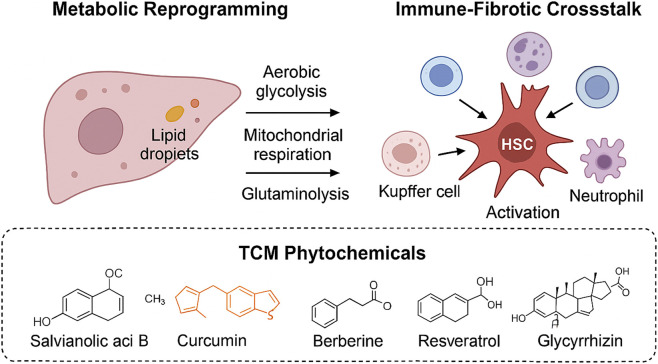
Schematic representation of the immunometabolic axis in liver fibrogenesis, illustrating the key metabolic reprogramming events in hepatic stellate cells (HSCs) including lipid droplet loss and a shift to aerobic glycolysis, glutaminolysis, and altered mitochondrial respiration alongside their critical crosstalk with immune cells (e.g., Kupffer cells and neutrophils) that sustains a cycle of inflammation and fibrosis, and highlighting selected plant-derived metabolites isolated from medicinal plants used in TCM (salvianolic acid B, curcumin, berberine, resveratrol, and glycyrrhizin) that intervene in these integrated pathways.

These metabolic abnormalities are deeply intertwined with immune-fibrotic interactions within the hepatic microenvironment. Kupffer cells and infiltrating macrophages release inflammatory cytokines, e.g., TNF-*α*, IL-1*β*, and TGF-*β* that intensify metabolic stress and accelerate HSC activation (Pradere et al., 2013) *via* promoting glycolysis, mitochondrial ROS production, and PI3K–Akt–mTOR signaling (Kostallari et al., 2025; Park et al., 2023; Bonnardel et al., 2019; Desai et al., 2022). Macrophage polarizes into M1 and M2 phenotypes. M1 macrophages propagate inflammation and oxidative stress while M2 macrophages facilitate matrix deposition and tissue remodeling through impairing mitochondrial function (Lee et al., 2020). T-cell subsets also shape fibrotic paths, with Th17 cells driving pro-fibrotic inflammation and regulatory T cells exerting anti-fibrotic restraint (Guo et al., 2021; Guan et al., 2025; Fan et al., 2023) (Figure 1). Conventional antifibrotic drug development has limitations within this complex landscape. Single-target agents have struggled to disrupt the complex metabolic, inflammatory and fibrogenic circuits and many candidates show insufficient efficacy or intolerable toxicity. In contrast, plant-derived metabolites used in traditional medical systems have been investigated for their potential to modulate multiple signaling pathways. However, the extent to which these multi-target effects translate into clinically meaningful antifibrotic outcomes remains uncertain.

This review critically evaluates the role of plant metabolites in modulating the immunometabolic axis of hepatic stellate cells. TCM primarily employs complex botanical preparations rather than isolated metabolites. However, characterization of individual metabolites provides an opportunity to identify molecular mechanisms that may contribute to the pharmacological effects of these botanical drugs. Accordingly, this review focuses on selected plant-derived metabolites that have been isolated from medicinal plants used in TCM while recognizing that these purified metabolites do not represent the complete therapeutic interventions used in traditional practice. A comprehensive literature search was conducted in PubMed and Web of Science using relevant keywords such as ‘liver fibrosis’, ‘hepatic stellate cells’, ‘extracellular matrix’, ‘immunometabolic reprogramming’, ‘Kupffer cells’, ‘macrophages’, ‘T cells’ with metabolites including ‘salvianolic acid B’, ‘curcumin’, ‘berberine’, ‘resveratrol’, ‘glycyrrhizin’, ‘puerarin’, ‘tetrandrine’, ‘oroxylin A’, and ‘schisandrin B’. Articles published in English up to March 2026 were considered. Additional references were identified through cross-referencing cited studies. Although this review primarily focuses on liver fibrosis, steatosis is discussed where relevant, as it represents an early and metabolically critical stage in the progression of metabolic dysfunction-associated liver disease and contributes to the immunometabolic alterations that drive fibrogenesis. It should be noted that the terms non-alcoholic fatty liver disease (NAFLD), metabolic dysfunction-associated fatty liver disease (MAFLD), metabolic dysfunction-associated steatotic liver disease (MASLD), and non-alcoholic steatohepatitis (NASH) are used throughout this review when referring to original studies, reflecting the nomenclature adopted in the respective publications at the time of reporting. This review complies with the four pillars of best practice in ethnopharmacology, including pharmacological validation, methodological transparency, relevance to traditional use and critical assessment of safety. While this review focuses on metabolites isolated from medicinal plants used in TCM, several metabolites discussed (e.g., curcumin and resveratrol) originate from or are also widely used in other traditional medical systems, including Ayurvedic and Unani medicine. This review therefore emphasizes their mechanistic roles within TCM without implying exclusivity of origin.

## 2 Selected plant-derived metabolites associated with medicinal plants used in TCM

**FIGURE 2:**
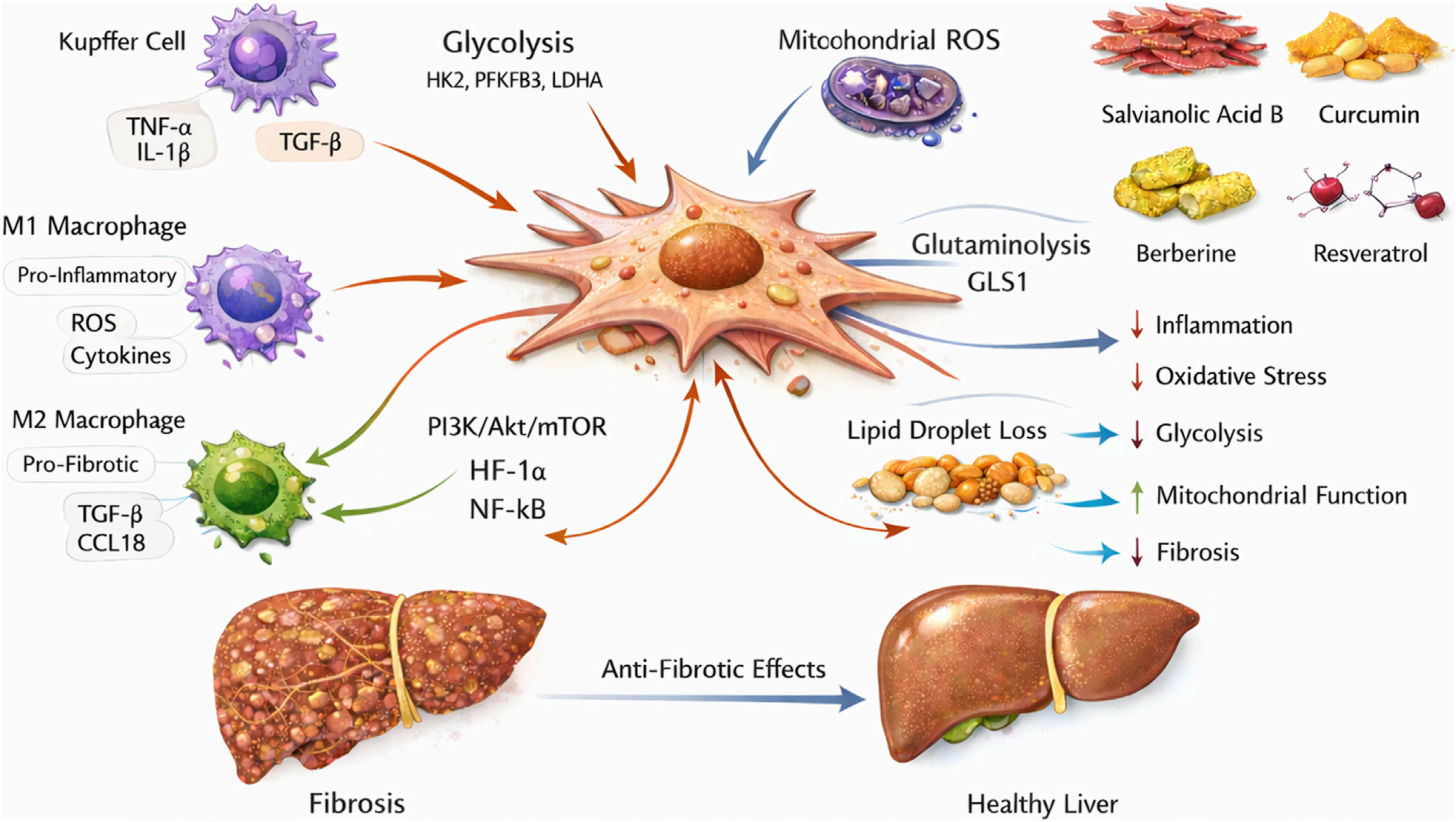
This schematic illustrates the central role of hepatic stellate cells (HSCs) in integrating immunometabolic signals that drive liver fibrosis. Pro-inflammatory inputs from Kupffer cells and M1 macrophages, including TNF-α, IL-1β, reactive oxygen species (ROS), and cytokines, promote HSC activation. Concurrently, M2 macrophages contribute to a pro-fibrotic environment through secretion of TGF-β and CCL18. Activated HSCs undergo metabolic reprogramming characterized by enhanced glycolysis (HK2, PFKFB3, LDHA), increased glutaminolysis (GLS1), mitochondrial ROS production, and activation of key signaling pathways such as PI3K/Akt/mTOR, HIF-1α, and NF-κB, collectively promoting extracellular matrix deposition and fibrosis progression. TCM-derived metabolites, including salvianolic acid B, curcumin, berberine, and resveratrol, have demonstrated antifibrotic activity in experimental models by targeting these immunometabolic pathways. These metabolites suppress inflammation, reduce oxidative stress, inhibit glycolysis, restore mitochondrial function, and improve lipid metabolism, thereby attenuating molecular and histological features associated with fibrosis in experimental models. The overall effect is the attenuation of fibrotic remodeling toward restoration of normal liver architecture and function.

TCM employs a variety of metabolites that are capable of influencing fundamental pathological circuits that drive liver fibrosis. Unlike single-pathway inhibitors, these metabolites often exert concerted effects across metabolic, mitochondrial, immune, and ECM-remodeling domains. In the following sections, nine leading metabolites are analyzed in depth, integrating mechanistic insights with *in-vitro* and *in-vivo* evidence and clinical observations if applicable. A comprehensive overview of the immunometabolic pathways involved in liver fibrosis and their modulation by metabolites used in TCM is illustrated in Figure 2.

### 2.1 Salvianolic acid B

Salvianolic acid B (SalB) is a major water-soluble polyphenol derived from *Salvia miltiorrhiza* Bunge (Lamiaceae), a botanical drug widely used in East Asian traditional medicine (Ho and Hong, 2011). It has demonstrated significant anti-fibrotic potential across multiple preclinical models including liver fibrosis (Liang et al., 2024), acting on interconnected metabolic, mitochondrial, immune and ECM-remodeling pathways. In rodent models of liver fibrosis, CCl_4_ and PDGF-induced fibrosis, SalB attenuated HSC activation, reduced extracellular matrix deposition and improved histopathological outcomes (Liu et al., 2023; Li et al., 2023). Li et al. demonstrated that intraperitoneal administration of sal B (purified metabolite) at 30 mg/kg/day inhibited HSC activation in LX-2 cells *in-vitro* at concentrations of approximately 50 μM, reducing *α*-SMA expression while pharmacological inhibition of UGCG using PDMP (IC_50_ = 62.8 μM) demonstrated dose-dependent suppression of HSC proliferation and fibrogenic markers. *In-vivo*, in a CCl_4_-induced liver fibrosis model with a 4-week treatment regimen, SalB reduced collagen deposition, hydroxyproline content and *α*-SMA expression (Li et al., 2023). SalB exerts pleiotropic effects by modulating key pro-fibrotic signaling pathways, including TGF-*β*/Smad, PDGF/PDGFR, Hedgehog, Wnt and MAPK cascades while reducing oxidative stress and inflammatory cytokine production (Liu et al., 2023; Wu et al., 2019; Tao et al., 2021; Yu et al., 2016). Wu et al. reported that Sal B, a purified metabolite, attenuated liver fibrosis in a diethylnitrosamine (DEN)-induced mouse model following intraperitoneal administration at 30 mg/kg/day. *In-vitro*, Sal B inhibited TGF-*β*1-induced activation of hepatic stellate cells, suppressing *α*-SMA and Collagen I expression through modulation of MAPK-mediated Smad2/3 signaling (Wu et al., 2019). Similarly, Tao et al. demonstrated that Sal B, a purified metabolite isolated from the roots of *S. miltiorrhiza* Bunge, protected against acute and chronic liver injury in CCl_4_-induced mouse models and oxidative stress in H_2_O_2_-treated hepatic cells. Sal B improved liver histology, reduced aminotransferase levels, attenuated oxidative stress and modulated TGF-*β*/Smad2 and Nrf2/HO-1 signaling by decreasing phosphorylation of Smad2 at both C-terminal and linker regions (p-Smad2C/L) while enhancing Nrf2 and HO-1 expression (Tao et al., 2021).

As stated in introductory paragraphs above, immunometabolic pathways are pivotal in modulating HSCs that prime the hepatocytes for liver fibrosis. In this context, SalB has been shown to modulate glycolysis-dependent processes in liver macrophages suggesting potential effects on metabolic reprogramming, although direct evidence in HSCs remains limited (Shi et al., 2010; Song et al., 2025; Hu et al., 2024; Zou et al., 2022). Similarly, SalB allows re-accumulation of retinoid-containing lipid droplets by inhibiting lipolytic enzymes that are otherwise upregulated during activation (Khomich et al., 2020). Concomitantly, evidence from Wang et al. indicates that purified salB protected against NASH in high-fat diet-induced SD rats. SalB was administered orally at 20 mg/kg/day and improved liver histology, enhanced mitochondrial morphology and respiratory function, increased Mfn2 expression, decreased NF-κB, cytochrome c (CytC), and caspase-3 levels, and attenuated oxidative stress and apoptosis (Wang et al., 2015). Immunologically, SalB downregulates TGF-*β*/Smad signaling, decreases Kupffer cell-derived inflammatory cytokines and attenuates macrophage recruitment, collectively weakening the immunometabolic loop that sustains fibrogenesis (Song et al., 2025; Khomich et al., 2020). *In-vitro*, it consistently downregulates *α*-SMA, collagen I, TIMP-1 and pro-inflammatory mediators in activated HSCs (Zhang W. et al., 2019). Moreover, in CCl_4_-induced hepatic fibrosis models in rats, salB (150 mg/kg) and its matrine salt (25–100 mg/kg) attenuate liver injury and fibrosis, reducing hydroxyproline and suppressing *α*-SMA and TGF-*β*1 expression. These effects are accompanied by improved antioxidant status and preservation of mitochondrial function supporting mitochondrial biogenesis and limiting fibrotic remodeling (Gao et al., 2012).

In Histopathological terms, SalB has demonstrated significant antifibrotic efficacy in preclinical models. *In-vivo* studies have shown that treatment with salB markedly reduces collagen deposition and fibrotic septa formation alongside improved hepatic architecture in hematoxylin and eosin (H&E) stained liver sections (Wu et al., 2019; Song et al., 2025; Tao et al., 2022; Fu et al., 2024). These histological improvements are accompanied by suppression of key profibrotic signaling pathways, including MAPK-mediated Smad2/3 phosphorylation (Wu et al., 2019), Hedgehog signaling (Tao et al., 2022) and glycolysis-dependent M1 macrophage polarization (Song et al., 2025). Additionally, salB has been reported to attenuate hepatocyte ferroptosis and extracellular matrix remodeling further contributing to the preservation of liver histoarchitecture (Fu et al., 2024). In conclusion, these effects position SalB as a modulator of the immunometabolic axis, linking metabolic reprogramming in HSCs and macrophages with suppression of pro-fibrotic immune signaling.

Despite consistent antifibrotic effects observed in preclinical models, several limitations constrain the interpretation and translational relevance of SalB. First, the majority of studies rely on rodent models (e.g., CCl_4_ or bile duct ligation), which incompletely recapitulate the complexity and chronicity of human liver fibrosis. Second, while multiple signaling pathways have been reported to be modulated by SalB, these findings are largely associative and causal relationships between specific metabolic targets and antifibrotic outcomes remain inadequately defined. Third, clinical evidence remains sparse and is largely derived from studies of complex botanical preparations rather than purified SalB, making it difficult to attribute observed effects specifically to this metabolite. Well-designed studies incorporating pharmacokinetic analysis, standardized dosing and clinically relevant endpoints are required to validate its translational potential.

### 2.2 Curcumin

Curcumin, the principal bioactive of *Curcuma longa* L. (Zingiberaceae) (El-Saadony et al., 2025), has a long history of use in Ayurvedic and Unani medicine and is also incorporated into TCM formulations. It directly interferes with metabolic reprogramming characteristic of activated HSCs. For instance, purified curcumin (20 μM) inhibited glycolytic flux in primary rat *HSCs* by downregulating key glycolytic enzymes including hexokinase (HK) and phosphofructokinase-2 (PFK2/PFKFB3) leading to reduced lactate production. These effects were mediated via AMPK activation as pharmacological inhibition with BML-275 reversed both metabolic suppression and antifibrogenic outcomes (Lian et al., 2016). Further, in LPS-induced septic mice and HL-1 cardiomyocytes stabilizes mitochondrial function by restoring membrane potential and reducing ROS while regulating mitochondrial fission–fusion dynamics *via* SIRT1-dependent inhibition of DRP1 translocation and activation of PGC-1*α*-mediated mitochondrial biogenesis (Hou et al., 2024). Through activation of Nrf2, purified curcumin enhances antioxidant defenses by inducing HO-1 and NQO1, providing secondary protection from oxidative injury (Ashrafizadeh et al., 2020).

Curcumin also exerts immunomodulatory effects. In LPS-stimulated macrophage models (J774A.1 cells), curcumin (2.5–20 μM) suppresses NF-κB signaling as reflected by reduced NF-κB p65 expression, leading to a dose-dependent decrease in pro-inflammatory cytokines such as TNF-*α*, IL-6, and IL-1*β* (Olivera et al., 2012; Liang et al., 2009). Further, a meta-analysis by Chu *et al.* discussing the intervention of curcumin on rodent models of hepatic fibrosis (Chu et al., 2024) demonstrated that curcumin consistently attenuates fibrosis severity across rodent models as evidenced by reductions in ALT/AST, collagen deposition (collagen I/III, hydroxyproline) and profibrotic mediators such as TGF-*β*1 and *α*-SMA, along with improved antioxidant status. In Mdr2^−/−^ mice, a model of sclerosing cholangitis, dietary curcumin (0.2% w/w in feed) attenuates disease progression by reducing cholestasis, ductular proliferation, and hepatic fibrosis, partly through suppression of cholangiocyte activation *via* PPARγ signaling and inhibition of portal myofibroblast proliferation *via* ERK1/2 pathways (Baghdasaryan et al., 2010). Another meta-analysis by Huang B.H. et al. on curcumin showed antifibrotic efficacy in metabolic liver disease models employing doses typically ranging from 50 to 300 mg/kg/day in rodents, leading to consistent reductions in fibrotic markers, liver enzymes, and oxidative stress (Huang et al., 2024). By modulating the Th17/Treg balance, curcumin also weakens pro-fibrogenic inflammatory environments (Ghoushi et al., 2024). Several clinical studies have tested curcumin in patients with metabolic or steatotic liver disease, yielding results consistent with modulation of immunometabolic pathways and partial amelioration of liver injury. For example, in a 12-month randomized controlled trial of patients with type 2 diabetes and MASLD, 1,500 mg/day curcumin significantly reduced systemic inflammatory markers (TNF-*α*, IL-1*β*, IL-6), oxidative stress, non-esterified fatty acids, body fat and hepatic steatosis and liver stiffness compared with placebo (Yaikwawong et al., 2025). In a shorter 12-week trial in patients with NAFLD, curcumin supplementation (1,500 mg/day) plus lifestyle advice was associated with reductions in liver fibrosis scores and decreased nuclear-factor-κB activity, suggesting dampening of inflammatory signaling pathways, although similar improvements occurred in the placebo group, highlighting the role of lifestyle intervention (Saadati et al., 2019). Similarly, a meta-analysis of randomized trials in NAFLD concluded that curcumin modestly improves liver enzymes, reduces steatosis and improves metabolic/lipid parameters, though evidence for fibrosis reversal remains limited (Huang et al., 2024). Moreover, a double-blind randomized trial of low-dose nano-curcumin (80 mg/day for 16 weeks) in adults with NAFLD-associated fibrosis (stage ≥ F2) observed significant reductions in ALT, AST, GGT and LDH, and a decline in the FIB-4 fibrosis index in the curcumin group, though the between-group difference in fibrosis or steatosis was not statistically significant (Gerami et al., 2025).

Curcumin has demonstrated notable antifibrotic effects in experimental models of liver disease, supported by histopathological evidence. *In-vivo* studies have shown that curcumin treatment significantly reduces collagen deposition and fibrotic septa formation along with improved hepatic architecture in hematoxylin and eosin (H&E)-stained liver sections. These histological improvements are accompanied by attenuation of steatohepatitis-associated fibrogenesis and modulation of the gut–liver axis, leading to reduced inflammatory signaling and hepatic stellate cell activation (Vizzutti et al., 2010). Furthermore, curcumin has been reported to regulate key profibrotic pathways and metabolic disturbances, thereby contributing to the preservation of liver histoarchitecture and suppression of fibrosis progression (Geng et al., 2024).

These studies suggest that curcumin can modulate metabolic dysfunction (insulin resistance, lipid/fat accumulation, oxidative stress) as well as inflammatory mediators and that such modulation may translate into improved liver stiffness/steatosis/fibrosis related biomarkers**.** However, the existing trials rarely include direct histological endpoints, mitochondrial functional assays or comprehensive immune–metabolic readouts. Though curcumin emerges as a promising immunometabolic modulator in human liver disease, further rigorous trials are needed to confirm its capacity to reverse established fibrosis and clarify its mechanisms.

### 2.3 Berberine

Berberine, an isoquinoline alkaloid found in several medicinal plants including *Coptis chinensis* Franch (Ranunculaceae) (Mirhadi et al., 2018) and related species is a powerful regulator of metabolic flux in liver fibrosis. Berberine’s capacity to reshape cellular immunometabolism has been increasingly recognized across inflammatory and metabolic disease models. For instance, Cheng *et al.* demonstrated that purified berberine markedly alleviates collagen-induced arthritis in mice by reprogramming macrophage metabolism, for example, restoring AMPK activation and suppressing mTORC1-driven glycolytic polarization, thereby shifting macrophages toward an anti-inflammatory phenotype (Cheng et al., 2023). Earlier, Yin et al. showed in insulin-resistant hepatocyte models (L6 myotubes and primary hepatocytes) that berberine (5–20 μM) enhances glucose utilization by inducing glycolysis through AMPK activation, highlighting its ability to redirect metabolic flux under insulin-resistant conditions (Yin et al., 2008). Complementing these findings, the comprehensive mechanistic review by Garcia and Shaw clarified how AMPK functions as a central cellular energy sensor that restores metabolic balance under stress conditions. This framework helps explain why AMPK activation is a consistent mechanistic axis through which berberine exerts both metabolic and immunoregulatory benefits (Garcia and Shaw, 2017). These studies imply berberine’s dual ability to modulate immune cell polarization and hepatic energy metabolism through AMPK-centered pathways relevant to chronic inflammatory states such as liver fibrosis.

Berberine has been shown in preclinical models to blunt innate immune activation in the injured liver by targeting the TLR4/MyD88/NF-κB signaling cascade. In a high-fat/high-fructose diet–induced NASH mouse model (C57BL/6 mice) Wang *et al.* treated animals with berberine at 100 mg/kg/day and reported decreased hepatic expression of TLR4 and MyD88, reduced phosphorylation and nuclear translocation of NF-κB p65 and lower transcription of NF-κB target cytokines (TNF-*α*, IL-6, IL-1*β*), consistent with direct suppression of the TLR4/NF-κB axis (Wang et al., 2021). Mechanistically, cell and molecular studies demonstrated that berberine interferes with the interaction between TLR4 and its adapter MyD88, preventing downstream NF-κB activation and M1 polarization in macrophages. This *in-vitro* finding explains the reduced proinflammatory signaling seen *in-vivo* (Gong et al., 2019). Consistent with pathway inhibition, multiple studies measured immune-cell trafficking and found that berberine treatment lowered hepatic infiltration of F4/80^+^ macrophages and MPO^+^ neutrophils and diminished chemokine expression (for example, CCL2), thereby reducing the cellular drivers of fibrogenic inflammation (Wang et al., 2021). Importantly, several studies on liver reported that the net effect of TLR4/NF-κB suppression was an attenuation of Kupffer cell activation. Isolated Kupffer cells from treated animals released less TNF-*α* and IL-1*β ex vivo*, and whole-liver cytokine assays showed commensurate declines in circulating and hepatic proinflammatory cytokines (Wang et al., 2021). These molecular and cellular changes translated into reduced hepatocellular injury, lower serum ALT/AST, decreased steatosis/inflammation scores on histology and in longer protocols, attenuation of collagen deposition and HSCs activation that links TLR4/NF-κB inhibition by berberine to downstream anti-fibrotic effects (Gupta et al., 2024; Ali and Datusalia, 2024). Further, primary studies support a model in which berberine interrupts upstream TLR4 signaling (including competitive or allosteric interference with TLR4–MyD88), thereby preventing NF-κB-driven cytokine storms, limiting neutrophil/macrophage recruitment and reducing Kupffer cell-mediated hepatic inflammation (Wang et al., 2020).

Histological evaluations in experimental models indicate that berberine can mitigate structural liver damage with reductions in collagen accumulation and partial restoration of normal hepatic architecture observed in Masson’s trichrome and H&E-stained sections. Mechanistically, these effects have been linked to redox-mediated induction of ferroptosis in activated hepatic stellate cells (Yi et al., 2021). Complementary clinical and preclinical evidence further suggests that berberine exerts beneficial effects during early, pre-fibrotic stages of metabolic liver disease where improvements in steatosis and inflammatory histological features have been reported (Geng et al., 2024). These steps are plausibly responsible for the metabolite’s anti-inflammatory and anti-fibrotic actions in preclinical liver disease models (Gong et al., 2019). In summary, berberine emerges as a multifaceted modulator of hepatic immunometabolism, exerting anti-inflammatory and anti-fibrotic effects through coordinated regulation of AMPK signaling, suppression of the TLR4/MyD88/NF-κB axis and modulation of immune cell polarization and trafficking. These integrated actions translate into improved metabolic homeostasis, reduced hepatic injury, and attenuation of fibrogenic progression in preclinical models. However, the current evidence is largely derived from *in-vitro* systems and animal studies with limited well-powered clinical validation. Variability in dosing regimens, bioavailability constraints and incomplete mechanistic resolution remain important limitations. Future studies integrating pharmacokinetic optimization and rigorous clinical trials are required to substantiate berberine’s therapeutic potential in human liver fibrosis.

### 2.4 Resveratrol

Resveratrol, a stilbene metabolite found in multiple plant sources including *Polygonum cuspidatum* Siebold and Zucc. (Polygonaceae) (Koushki et al., 2018), exerts multipronged effects on HSC energy metabolism and inflammatory signaling. Resveratrol exerts hepatoprotective effects largely through activation of the SIRT1–PGC-1*α* axis, promoting mitochondrial biogenesis and enhancing fatty-acid oxidation in high-fat diet–induced obese C57BL/6J mice treated with purified resveratrol at 100–400 mg/kg/day (Lagouge et al., 2006). Lagouge *et al.* showed that resveratrol activates SIRT1 and deacetylates PGC-1*α*, leading to increased mitochondrial content and improved oxidative capacity (Lagouge et al., 2006). Similarly, in experimental fatty-liver and fibrosis models in C57BL/6 mice, resveratrol administered at doses of 100–200 mg/kg/day of purified resveratrol upregulated SIRT1/PGC-1*α* signaling, increased markers of mitochondrial function and *β*-oxidation and reduced steatosis and oxidative stress, thereby lowering profibrogenic stimuli (Ajmo et al., 2008). Importantly, several studies have linked SIRT1 activation by resveratrol to direct effects on HSCs. For instance, one study found that resveratrol promotes a quiescence-like phenotype and suppresses HSC activation at least in part by modulating PPARγ/SIRT1 balance, inducing autophagy/apoptosis and reducing collagen synthesis (de Souza et al., 2015). This data support a mechanistic model in which resveratrol restores mitochondrial homeostasis and fatty-acid oxidation while antagonizing HSC activation through SIRT1-dependent deacetylation of PGC-1*α* (Lagouge et al., 2006).

Resveratrol also mitigates profibrotic metabolic reprogramming by down-regulating hypoxia-inducible factor-1*α* (HIF-1*α*) signaling and consequential lactate accumulation thereby constraining glycolysis-driven profibrotic cues. For instance, Zhang et al. reported that resveratrol reduces HIF-1*α* and VEGF expression and limits hypoxia-induced glucose uptake and lactate production in hepatic/cancer cell models supporting a mechanism in which HIF-1*α* correction lowers lactate-dependent signaling (Zhang et al., 2014). Mechanistically, resveratrol’s suppression of intracellular ROS contributes to reduced HIF-1*α* stabilization and downstream glycolytic enzyme expression, reinforcing metabolic reprogramming away from aerobic glycolysis (Jung et al., 2013). Consistent with this, in ethanol-fed C57BL/6J mouse models of alcoholic fatty liver disease, resveratrol administered at 100 mg/kg/day restored hepatocyte mitochondrial function, limited mitochondrial ROS production, downregulated HIF-1*α* expression and improved cellular redox homeostasis and resistance to oxidative injury (Ma et al., 2017). At the level of HSCs, these redox- and metabolism-modulating actions translate into suppression of activation and promotion of quiescence-like phenotypes. Several *in-vitro* and *in-vivo* studies report decreased HSC proliferation, reduced collagen gene expression and increased markers of autophagy/apoptosis following resveratrol treatment (Ma et al., 2022). Similarly, in a rat model of warm hepatic ischemia-reperfusion injury in SD rats resveratrol administered intravenously at 20 mg/kg prior to ischemia effectively attenuated HIF-1*α* and VEGF expression, limited ROS generation, and improved hepatocellular redox balance (Zhang et al., 2014).

Resveratrol has been shown to attenuate activation of hepatic innate immune cells including Kupffer cells, as a result dampening inflammatory signalling that drives fibrogenesis. In experimental cholestatic liver injury and toxin-induced fibrosis models, resveratrol treatment resulted in decreased hepatic levels of pro-inflammatory cytokines such as IL-6 and TNF-*α*, along with reduced NF-κB activation which is consistent with suppression of Kupffer cell activation and inflammatory output (Ma et al., 2022). Downstream, resveratrol reduces signalling through the IL-6/STAT3 axis, a pathway known to promote survival and activation of myofibroblastic fibrogenic cells. Although direct STAT3 phosphorylation data are more limited, the suppression of IL-6 and upstream NF-κB suggests attenuated STAT3 activation in the fibrotic environment. At the level of HSCs, resveratrol inhibits proliferation, reduces expression of activation markers including *α*-smooth muscle actin (*α*-SMA) and decreases ECM deposition, as shown in cell-culture treated HSC models (Zhang et al., 2016). In addition to inhibiting activation, resveratrol induces apoptosis and/or autophagy in activated HSCs contributing further to a quiescent or deactivated phenotype (Ma et al., 2022). *In-vivo*, these cellular effects translate into significantly slower progression of fibrosis across diverse liver injury models with reductions in collagen deposition, ECM accumulation, and fibrotic area (Li et al., 2021).

Histopathological findings indicate that resveratrol confers protective effects against liver injury, as reflected by improved tissue architecture and reduced inflammatory infiltration in H&E-stained sections of high-fat diet-induced models. These structural improvements are accompanied by attenuation of oxidative stress and preservation of gut–liver axis integrity, which collectively limit fibrogenic progression (Yasmin et al., 2025a). In addition, evidence from related experimental models highlights the capacity of resveratrol to mitigate oxidative stress–driven tissue damage and inflammation, further supporting its role in preserving organ histoarchitecture (Yasmin et al., 2025b).

Conclusively, the preclinical and clinical evidence supports a model in which resveratrol attenuates Kupffer cell–mediated inflammation, suppresses IL-6–driven fibrogenic signalling, deactivates HSCs (*via* reduced *α*-SMA/ECM synthesis and induction of apoptosis/autophagy), and improves metabolic derangements which results in slowing or reversing liver fibrosis progression. A recent systematic review and meta-analysis of preclinical studies confirms these findings, noting consistent reductions in fibrotic markers, inflammatory cytokines and oxidative stress in resveratrol-treated animals (Luo et al., 2025).

Despite promising preclinical evidence, the therapeutic translation of resveratrol is limited by several factors. Most studies rely on rodent models or *in-vitro* systems, which may not fully recapitulate human liver disease pathophysiology. Additionally, resveratrol exhibits poor oral bioavailability and variable pharmacokinetics and the effective doses used in animal studies are often much higher than those safely achievable in humans. Long-term safety, optimal dosing regimens and efficacy in well-controlled clinical trials remain to be established.

### 2.5 Puerarin

Puerarin, an isoflavone derived from *Pueraria lobata* (Willd.) Ohwi (Fabaceae), appears to restrain liver fibrogenesis through coordinated effects on cellular metabolism, ECM production, and inflammation. In toxin- and chemically induced fibrosis models (e.g., CCl_4_, DMN, TAA), administration of puerarin significantly lowered markers of hepatic injury (ALT/AST), reduced collagen deposition and hydroxyproline content, and downregulated profibrotic proteins such as *α*-SMA, collagen I and fibronectin which indicate suppression of HSC activation and ECM accumulation (Li et al., 2013). In mechanism, the anti-fibrotic effect has been linked not only to inhibition of the classical fibrogenic TGF-*β*1/Smad signaling pathway but also to modulation of metabolic-signaling cascades. For instance, purified puerarin metabolite treatment suppressed phosphorylation of mTOR and Akt while altering mTOR complex components, suggesting attenuation of PI3K/Akt/mTOR signaling in injured liver tissue. In CCl_4_–induced liver injury in SD rats, puerarin was administered intraperitoneally at 100 mg/kg/day for 7 days, resulting in decreased p-mTOR and p-Akt levels and modulation of mTOR complex proteins in hepatic tissue (Zhou et al., 2018). In models of metabolic liver disease, puerarin ameliorated steatosis, improved lipid homeostasis and reduced oxidative stress and inflammatory cytokine levels (Li et al., 2024). These metabolic improvements suggest restoration of more quiescent, non-activated HSC/hepatocyte phenotypes, possibly via enhanced mitochondrial function and reduced lipotoxicity.

Concomitantly, puerarin exerts immunomodulatory and anti-inflammatory effects. In CCl_4_-induced fibrosis, puerarin decreased activation of inflammatory pathways, increased antioxidant defenses and lowered markers of oxidative stress (MDA), consistent with attenuation of inflammation and hepatocellular injury (Li et al., 2013). Similarly, in a MAFLD model established in male C57BL/6J mice by 12 weeks of high-fat diet feeding followed by streptozotocin injection to induce hyperglycemia, puerarin administered by oral gavage at 200 mg/kg body weight three times per week for 4 weeks boosted hepatic SIRT1 and nuclear Nrf2 expression, inhibited ferroptosis, suppressed inflammatory cytokines, and improved insulin sensitivity, liver enzyme levels, and lipid profiles (Yang et al., 2023).

Morphological assessments from experimental fibrosis models indicate that puerarin administration leads to marked improvement in liver histoarchitecture with diminished collagen accumulation and reduced fibrotic bridging observed alongside restoration of hepatocellular integrity in H&E staining. These structural changes are associated with inhibition of key profibrotic mediators, including PARP-1 and ERK1/2 signaling pathways, which contribute to attenuation of hepatic stellate cell activation (Wang et al., 2016; Li et al., 2019). The convergence of histological and molecular findings underscores the capacity of puerarin to counteract fibrogenic remodeling in chemically induced liver injury models.

Despite these encouraging preclinical findings, the translational potential of puerarin is limited by several factors. Most studies have been conducted in rodent or *in-vitro* models which may not fully reflect human liver disease. Additionally, the effective doses used in animals are relatively high and may not be achievable or safe in humans and pharmacokinetic data, including bioavailability and metabolism, remain limited. Long-term safety, optimal dosing regimens, and efficacy in well-controlled clinical trials have yet to be established.

### 2.6 Glycyrrhizin

Glycyrrhizin (GL), a triterpenoid saponin derived from the root of Glycyrrhiza uralensis Fisch (Fabaceae)., has been extensively studied for its hepatoprotective, anti-inflammatory and anti-fibrotic properties. In a pivotal early study, GL was demonstrated to bind directly to HMGB1, the nuclear “alarmin” released by damaged hepatocytes and immune cells, thereby blocking HMGB1’s extracellular cytokine‐ and chemoattractant activities. For instance, GL reduced HMGB1-induced migration of fibroblasts by almost 50% at 50 μM, highlighting a specific suppression of HMGB1-mediated signalling rather than a general cytotoxic effect (Mollica et al., 2007). Concomitantly, *in-vitro* studies using murine macrophages in RAW264.7 cells treated with LPS showed that GL prevented the cytoplasmic translocation of HMGB1, inhibited its mRNA upregulation and suppressed downstream inflammatory signaling including p38 MAPK/AP-1 activation that leads to marked reductions in pro-inflammatory cytokines (Wu et al., 2015). In an *in-vivo* model of LPS-induced acute liver injury, GL treatment attenuated liver inflammation and decreased HMGB1 and apoptotic markers, effects that were at least partly mediated through suppression of the PI3K/mTOR pathway (Shen et al., 2020).

Beyond its immunomodulatory role, GL (and its active derivative, Glycyrrhetinic acid, GA) exert anti-fibrotic effects by directly acting on HSCs. In transgenic reporter mice carrying the type-I collagen promoter, both GL and GA significantly suppressed promoter activation and prevented collagen accumulation in a chronic fibrotic model induced by repeated CCl_4_ injections. Detailed *in-vitro* assays further revealed that GA inhibited nuclear accumulation of Smad3 in TGF-*β*1–activated HSCs, indicating blockade of canonical TGF-*β*/Smad signaling (Moro et al., 2008). More recently, a 2024 study demonstrated that GL upregulates the detoxifying enzyme AKR7A2 in both LX-2 HSCs and in murine livers, reducing oxidative stress and thereby suppressing HSC activation and proliferation (Wang et al., 2024). At the level of hepatocyte preservation, GL mitigates hepatocyte apoptosis in CCl_4_-induced fibrosis. In a classic rat model, administration of GL lowered ALT, AST, reduced collagen deposition, and decreased the proportion of TUNEL-positive hepatocytes (Liang et al., 2015). Further, GL suppressed activation of p53, lowered the Bax/Bcl-2 ratio, prevented cytochrome-c and Smac release from mitochondria and ultimately inhibited caspase-9 and caspase-3 activation, thereby blocking the mitochondrial (intrinsic) apoptotic cascade (Guo et al., 2013). Additionally, GL has been shown to antagonize HMGB1-induced hepatocyte apoptosis *via* inhibition of the p38 MAPK–dependent mitochondrial pathway (Gwak et al., 2012).

Tissue-level analyses demonstrate that glycyrrhizin and its active form, glycyrrhizic acid, exert protective effects against fibrotic remodeling. These improvements are accompanied by attenuation of inflammatory infiltration and suppression of profibrotic signaling cascades, including activation of the CUGBP1-mediated IFN-γ/STAT1/Smad7 axis (Guo et al., 2023). Evidence from systemic fibrosis models further supports its capacity to mitigate extracellular matrix accumulation and vascular alterations (Yamashita et al., 2017). This data presents the GL as a promising candidate for pharmacological manipulation for the treatment of liver fibrosis *via* targeting immunometabolic axis.

Although the therapeutic application of GL is limited by several factors. Most studies have been conducted in cell lines or rodent models, which may not fully reflect human liver fibrosis. Although it showed promising effects on metabolic disease targets, but its effects in key metabolic markers are yet to be evaluated, especially in liver fibrosis settings. Moreover, long-term safety, potential mineralocorticoid-related side effects, and robust clinical evidence for chronic liver disease remain to be established.

### 2.7 Tetrandrine

Tetrandrine, a bis-benzylisoquinoline alkaloid isolated from *Stephania tetrandra* S. Moore (Menispermaceae) exerts anti-fibrotic actions in the liver through coordinated effects on calcium signaling, mitochondrial metabolism and immune activation. Early experimental work showed that tetrandrine reduces HSCs proliferation and markers of activation in cultured HSCs while improving fibrosis endpoints in bile-duct-ligation models, indicating a direct effect on HSCs rather than only an indirect hepatoprotective action (Park et al., 2000). In mechanism, tetrandrine’s well-characterized blockade of voltage-gated and receptor-operated Ca^2+^ channels prevents pathological cytosolic Ca^2+^ overload in activated HSCs. This normally precipitates mitochondrial depolarization, excess reactive oxygen species (ROS) generation and the metabolic switch that sustains a fibrogenic phenotype. *In-vitro* investigations found that tetrandrine decreases *α*-SMA and collagen I expression, suppresses TGF-*β*1-driven signaling and upregulates inhibitory Smad7, consistent with direct blockade of canonical pro-fibrotic pathways in HSCs (Chen et al., 2005). Tetrandrine also modulates the inflammatory drivers of fibrosis. Several studies show it inhibits NF-κB activation in macrophages or Kupffer-like cells and reduces production of TNF-*α*, IL-1*β* and other pro-inflammatory mediators. In the study by Xu et al., *β*-glucan–activated murine and human macrophages were treated with tetrandrine at defined *in-vitro* concentrations, which inhibited NF-κB, ERK, and STAT3 activation and reduced TNF-*α* and IL-1*β* production (Xu et al., 2016). *In-vivo*, across thioacetamide (TAA), dimethylnitrosamine (DMN) and bile-duct-ligation models, tetrandrine treatment lowers serum aminotransferases, reduces liver hydroxyproline content and collagen deposition, improves histologic architecture and reduces portal pressure (Park et al., 2000; Hsu et al., 2007; Yin et al., 2007). Histopathological evidence further supports the anti-fibrotic efficacy of tetrandrine in experimental liver injury. In a carbon tetrachloride (CCl_4_)–induced fibrosis model in rats, chronic administration of tetrandrine (30 and 50 mg/kg) over 14 weeks markedly attenuated liver damage, as demonstrated by reduced steatosis, diminished fibrotic septa formation and preservation of hepatic architecture on light microscopy (Choi et al., 1999). Collectively these effects demonstrate both hepatoprotective and antifibrotic efficacy and provide future promise for fibrosis therapeutics.

The current evidence for tetrandrine is largely derived from *in-vitro* systems and rodent models, which may not fully translate to human liver fibrosis. Additionally, the doses used in experimental studies and the metabolite’s pharmacokinetic profile, including bioavailability and potential toxicity are not well defined for clinical use. Well-controlled clinical trials and long-term safety evaluations are therefore required to validate its therapeutic potential.

### 2.8 Oroxylin A

Oroxylin A (OA), an O-methylated flavone abundant in *Scutellaria baicalensis* Georgi (Lamiaceae) has emerged as a compound with reported immunometabolic effects in experimental systems. Mechanistic work in HSCs shows that OA suppresses the glycolytic program that sustains HSC activation. In human LX-2 cells and primary HSCs, OA downregulated glycolytic enzymes (HK2, PFK1, PKM2) and inhibited LDH-A activity, which translated into reduced glucose uptake, lactate production and a loss of glycolysis-dependent contractility, effects that were also observed after oral OA treatment in CCl_4_-fibrotic mice. This blockade of aerobic glycolysis deprives activated HSCs of the metabolic flux required for sustained myofibroblastic function and matrix remodeling (Wang et al., 2019). OA also modulates mitochondrial and lipid metabolism. Multiple studies indicate OA improves mitochondrial respiration and mitochondrial quality-control pathways, including PGC-1*α*/Mfn2 signaling (Kai et al., 2020) and SIRT3-dependent effects (Liu et al., 2024), stabilizes mitochondrial membrane potential and reduces ROS generation in hepatocytes and HSCs therefore shifting cells away from a glycolysis-dominant, pro-fibrotic phenotype toward enhanced fatty-acid oxidation and oxidative phosphorylation (Wei et al., 2015). From an immunologic perspective, OA exerts significant anti-inflammasome and anti-cytokine actions. OA suppresses NLRP3 inflammasome assembly and caspase-1 activation in macrophages, limiting IL-1*β* maturation. These anti-inflammasome effects have been reported across inflammatory models and are consistent with the reductions in hepatic IL-1*β*, TNF-*α* and overall inflammatory scores seen in OA-treated animals (Zhou et al., 2017). Crucially, these mechanistic data are supported by *in-vivo* antifibrotic efficacy. In CCl_4_ induced fibrosis, OA lowered serum transaminases, decreased hydroxyproline and collagen deposition, reduced *α*-SMA expression, and restored sinusoidal fenestration; genetic or pharmacologic perturbation of glycolytic nodes blunted OA’s benefits, supporting causality between glycolytic suppression and anti-fibrotic effects. In diet-induced NASH and steatohepatitis models, including high-fat diet–fed C57BL/6 mice, purified Oroxylin A administered at pharmacological doses (50–100 mg/kg/day) reduced hepatic lipid peroxidation, improved insulin sensitivity, and ameliorated systemic metabolic dysfunction, thereby linking its hepatoprotective and anti-fibrotic effects to a broader correction of immunometabolic disturbances (Wang et al., 2019).

Collectively, these findings position Oroxylin A as a potent immunometabolic modulator that attenuates liver fibrosis by suppressing glycolysis, restoring mitochondrial function, and dampening inflammatory signaling, thereby disrupting key drivers of HSC activation and fibrogenesis. However, the current evidence is largely derived from *in-vitro* systems and rodent models, limiting direct clinical translatability. Clinical studies are required to validate its therapeutic efficacy and optimize its use in human liver disease.

### 2.9 Schisandrin B

Schisandrin B (Sch B), a dibenzocyclooctadiene lignan from *Schisandra chinensis* (Turcz.) Baill (Schisandraceae) has robust evidence supporting its role as a mitochondrial antioxidant and modulator of redox-sensitive fibrogenic signalling. Early mechanistic studies demonstrated that purified schisandrin B enhances mitochondrial antioxidant defenses and heat-shock protein responses, thereby increasing mitochondrial resilience to injury. In CCl_4_-induced hepatotoxicity models in mice, Sch B pretreatment protected hepatic mitochondria by reducing Ca^2+^-induced permeability transition, mitochondrial ROS production and cytochrome c release. Complementary studies in rats showed that chronic Sch B administration at 10 mg/kg/day for 15 days improved mitochondrial antioxidant status and enhanced resistance to Ca^2+^-induced mitochondrial permeability transition, alongside induction of heat-shock proteins (Hsp25/70), collectively supporting its role in preserving mitochondrial integrity under oxidative stress conditions in liver fibrosis (Chiu et al., 2007; Chiu et al., 2006). These mitochondrial effects translate into preserved membrane potential and improved ATP synthesis, which limit ROS-driven activation of JNK/TGF-*β* axes in parenchymal and stromal cells (Chiu et al., 2007). Mechanistically, Sch B activates the Keap1–Nrf2–ARE axis and upregulates downstream antioxidant enzymes (HO-1, NQO1, GCLC), an effect confirmed by metabolite/Keap1 binding studies and Nrf2 reporter assays. Nrf2 induction both reduces lipid peroxidation in hepatocytes and blunts redox-dependent HSC activation, linking Sch B’s antioxidant signaling to suppression of fibrogenic programming. In macrophages and Kupffer-like cells, Sch B inhibits NF-κB and pro-inflammatory mediator release (TNF-*α*, IL-1*β*, NO), thereby decreasing the paracrine inflammatory cues that drive HSC transdifferentiation (Yao et al., 2018; Chen et al., 2017). *In-vitro*, Schisandrin B reverses HSC activation markers (*α*-SMA, COL1A1), promotes apoptosis of activated HSCs, and suppresses TGF-*β*/Smad signaling supporting a direct anti-fibrotic action on the primary ECM-producing cells. *In-vivo*, in CCl_4_-induced hepatotoxicity models in mice, Sch B administered as a pretreatment at pharmacologically active doses (25–100 mg/kg) reduced mitochondrial Ca^2+^ overload, ROS production, and cytochrome c release by limiting Ca^2+^-induced mitochondrial permeability transition, thereby preserving mitochondrial integrity. Complementary transcriptomic and functional analyses in CCl_4_-treated rodents further demonstrated reduced collagen deposition, hydroxyproline content, inflammatory infiltration, and serum transaminases alongside restoration of mitochondrial gene signatures and antioxidant pathways (Zhang H. et al., 2019).

Collectively, Schisandrin B emerges as a potent mitochondrial-targeted antioxidant that mitigates liver fibrosis through coordinated regulation of redox homeostasis, suppression of pro-inflammatory signaling, and direct inhibition of HSC activation. Its ability to stabilize mitochondrial function, activate Nrf2-dependent antioxidant pathways, and attenuate TGF-*β*–driven fibrogenesis highlights a multifaceted therapeutic potential. However, the current evidence is predominantly derived from *in-vitro* systems and rodent models, limiting direct clinical translatability. In addition, variability in dosing regimens, incomplete pharmacokinetic characterization and insufficient long-term safety and human efficacy data remain key limitations that warrant further investigation.

## 3 Critical appraisal of current evidence

The available evidence suggests that several plant-derived metabolites associated with medicinal plants used in TCM can influence immunometabolic pathways implicated in liver fibrogenesis. A notable strength of the current literature is the recurrent observation that structurally diverse metabolites converge on a limited number of biologically relevant pathways, including TGF-β/Smad, NF-κB, PI3K–Akt–mTOR, HIF-1α, mitochondrial oxidative stress, and metabolic processes regulating glycolysis and lipid homeostasis. Across multiple experimental models, these compounds are consistently associated with reduced hepatic stellate cell activation, decreased extracellular matrix deposition, attenuation of inflammatory signaling, and improvement of histological fibrosis markers. Such convergence across independent studies provides biological plausibility for the immunometabolic mechanisms discussed in this review.

However, important limitations substantially constrain interpretation of the available evidence. First, most data originate from *in-vitro* experiments and rodent models, particularly carbon tetrachloride-, thioacetamide-, and bile duct ligation-induced fibrosis. Although these models reproduce selected features of fibrogenesis, they do not fully capture the complexity, chronicity, and heterogeneity of human liver fibrosis. Second, many studies rely heavily on changes in molecular markers and signaling pathways, whereas direct mechanistic evidence linking specific metabolic alterations to fibrosis regression remains limited. Consequently, causal relationships are often inferred rather than definitively established.

Additional challenges relate to translational applicability. Many metabolites exhibit poor oral bioavailability, variable pharmacokinetic profiles, and limited tissue-specific delivery, raising uncertainty regarding whether effective experimental concentrations can be achieved in humans. Furthermore, substantial heterogeneity exists among studies with respect to animal models, treatment duration, dosing regimens, outcome measures, and fibrosis stage, complicating direct comparison and synthesis of findings. Clinical evidence remains particularly limited, and few studies have incorporated histological endpoints, standardized biomarkers of immunometabolic activity, or long-term assessments of fibrosis progression or regression.

Importantly, several beneficial effects reported in experimental studies may reflect prevention of fibrogenic signaling or attenuation of ongoing injury rather than true reversal of established fibrosis. This distinction is clinically relevant because advanced fibrosis and cirrhosis involve extensive extracellular matrix crosslinking, altered tissue architecture, and persistent cellular reprogramming that may not be readily reversible. Therefore, while the current body of evidence provides a compelling rationale for further investigation of these metabolites as mechanistic probes and potential therapeutic leads, their clinical antifibrotic efficacy remains insufficiently established. Future research should prioritize rigorous mechanistic validation, standardized experimental frameworks, pharmacokinetic characterization, and adequately powered clinical studies with clinically meaningful endpoints.

## 4 Future directions

The growing evidence of HSC metabolic reprogramming and immune-fibrotic cross-talk provides a framework for the next-generation of antifibrotic therapeutics. While metabolites isolated from medicinal plants used in TCM have demonstrated efficacy in preclinical models and limited clinical studies, several avenues remain to be explored to maximize their translational potential. First, the integration of systems pharmacology with metabolomics and single-cell transcriptomics could unravel the full spectrum of metabolic-immune nodes modulated by individual metabolites. By delineating how selected metabolites have been reported to influence glycolysis, glutaminolysis, FAO, and redox signaling in HSCs while shaping macrophage and T cell phenotypes, researchers can prioritize metabolites or their combinations with complementary pharmacological effects and minimal off-target toxicity.

Artificial intelligence (AI)-assisted drug discovery and systems-level analytical approaches may further accelerate the development of metabolites-based antifibrotic therapies. Machine learning algorithms can integrate large pharmacological, transcriptomic, metabolomic, and chemical datasets to predict novel targets, optimize compound combinations, and identify patient subgroups most likely to benefit from specific interventions. Similarly, network pharmacology approaches are particularly relevant to Traditional Chinese Medicine because they enable characterization of multi-component, multi-target interactions and facilitate identification of combinations with complementary mechanisms acting on interconnected immunometabolic pathways. Integration of single-cell transcriptomics, spatial omics, proteomics, and metabolomics datasets may further reveal previously unrecognized regulatory nodes within hepatic stellate cells and the surrounding immune microenvironment, thereby supporting rational design of precision antifibrotic therapies targeting the immunometabolic axis.

Second, combination strategies with existing Western therapies, such as FXR agonists, PPAR modulators, GLP-1 analogues, or antioxidants, could be systematically evaluated. Preclinical models suggest that metabolites isolated from medicinal plants used in TCM warrant investigation in combination with existing therapies by correcting HSC metabolic defects and mitigating inflammatory amplification loops, therefore allowing lower doses of synthetic agents and reducing adverse effects. Third, advances in targeted delivery systems, including nanoparticle-based carriers, liposomes, and liver-specific prodrugs, could enhance the bioavailability and tissue specificity of selected plant-derived metabolites associated with herbs used in TCM, overcoming the pharmacokinetic limitations that have historically constrained clinical translation.

Furthermore, rigorous, well-designed clinical trials are urgently needed to confirm the mechanistic insights derived from *in-vitro* and animal studies. Standardization of metabolites purity, dosing, and treatment regimens will be critical to ensure reproducibility and comparability across studies. Beyond conventional endpoints such as liver enzymes and imaging, incorporation of biomarkers reflecting HSC metabolic status, immune cell profiles, and ECM remodeling will provide mechanistic validation of clinical efficacy.

## 5 Conclusion

Liver fibrosis develops through a complex interplay of sustained hepatocellular injury, metabolic reprogramming of HSCs, and chronic inflammatory signaling from resident and infiltrating immune cells. The close association between steatosis and fibrosis further highlights the importance of addressing early metabolic dysfunction to reduce the risk of fibrotic progression. Selected plant-derived metabolites associated with herbs used in TCM may provide useful leads for future antifibrotic research, particularly because several of these compounds affect more than one biological pathway at the same time.

Compounds such as salvianolic acid B, curcumin, berberine, resveratrol, puerarin, glycyrrhizin, tetrandrine, oroxylin A, and schisandrin B have been reported to influence HSC glycolysis, glutaminolysis, mitochondrial function, lipid metabolism, and inflammatory signaling, including macrophage–HSC crosstalk. However, the current evidence is uneven across compounds and is strongest in preclinical models. In these studies, the compounds are associated with reduced HSC activation, decreased extracellular matrix deposition, and partial restoration of a quiescent cellular phenotype, but these findings do not yet establish clinical antifibrotic efficacy.

Among the compounds reviewed, curcumin, resveratrol, berberine, and glycyrrhizin have the most consistent translational signals, although even for these agents the available clinical evidence remains limited and often indirect. For other compounds, including salvianolic acid B, tetrandrine, oroxylin A, and schisandrin B, the evidence remains largely preclinical and mechanistically incomplete (Table 1). Overall, the available data suggest that these metabolites may serve as useful chemical leads for antifibrotic drug discovery, but their therapeutic value in human liver fibrosis remains to be confirmed in well-designed studies.

**TABLE 1:** Summary of key traditional Chinese medicine metabolites targeting the immunometabolic axis in liver fibrosis, including main molecular pathways, experimental models, and antifibrotic effects.

Compound	Main source	Key molecular targets/pathways	Experimental evidence	Level of evidence	Main antifibrotic effect
Salvianolic acid B	*Salvia miltiorrhiza*	TGF-β/Smad, PDGFR, Wnt, MAPK, NF-κB, Akt-mTOR, mitochondrial ROS, glycolysis-related effects	CCl_4_ and PDGF fibrosis models; HSC studies; macrophage polarization studies; human primary HSCs	Preclinical (*in-vitro* and animal studies)	Reduces HSC activation, ECM deposition, inflammation and mitochondrial dysfunction
Curcumin	*Curcuma longa*	HK2, PFKFB3, AMPK, Nrf2/HO-1/NQO1, NF-κB, Th17/Treg balance, mitochondrial dynamics	*In-vitro* HSC studies; CCl_4_, TAA, cholestatic, and NASH models; clinical trials in MASLD/NAFLD	Clinical evidence available	Suppresses glycolysis, oxidative stress, inflammation and HSC activation
Berberine	*Coptis chinensis*	AMPK, mTORC1, TLR4/MyD88/NF-κB, macrophage polarization, Kupffer cell activation	Mouse steatohepatitis models; macrophage and immune-cell studies; liver injury/fibrosis models	Preclinical (*in-vitro* and animal studies)	Lowers inflammatory signaling, immune-cell recruitment and downstream fibrotic progression
Resveratrol	*Polygonum cuspidatum*	SIRT1/PGC-1α, HIF-1, ROS, IL-6/STAT3, NF-κB, autophagy/apoptosis, FAO	*In-vitro* HSC studies; fatty liver and fibrosis animal models; some clinical data in metabolic liver disease	Clinical evidence available	Restores mitochondrial homeostasis and reduces HSC activation and collagen production
Puerarin	*Pueraria lobata*	TGF-β1/Smad, PI3K/Akt/mTOR, Nrf2, SIRT1, lipid metabolism, ferroptosis, NF-κB	CCl_4_ fibrosis models; experimental liver injury; MASLD/NAFLD models	Preclinical (animal studies)	Improves lipid metabolism, inflammation, oxidative stress and fibrosis markers
Glycyrrhizin	Licorice	HMGB1, p38 MAPK/AP-1, PI3K/mTOR, TGF-β/Smad3, AKR7A2, mitochondrial apoptosis pathways	RAW264.7 macrophages; LPS acute liver injury; CCl4 fibrosis; LX-2/HSC studies	Preclinical (*in-vitro* and animal studies)	Blocks HMGB1-driven inflammation and directly suppresses HSC activation and fibrosis
Tetrandrine	*Stephania tetrandra*	Calcium signaling, TGF-β1/Smad, Smad7, NF-κB, ERK, STAT3, mitochondrial ROS	Cultured HSCs; bile duct ligation, TAA, and DMN fibrosis models	Preclinical (*in-vitro* and animal studies)	Reduces HSC proliferation, collagen deposition, inflammatory signaling and portal fibrosis
Oroxylin A	*Scutellaria baicalensis*	Glycolytic enzymes HK2, PFK1, PKM2, LDH-A, PGC-1/Mfn2, SIRT3, NLRP3 inflammasome	LX-2 cells, primary HSCs, CCl_4_-fibrotic mice, NASH/steatohepatitis models	Preclinical (*in-vitro* and animal studies)	Inhibits aerobic glycolysis, improves mitochondrial function and reduces inflammasome-driven inflammation
Schisandrin B	*Schisandra chinensis*	Keap1/Nrf2/ARE, HO-1, NQO1, GCLC, NF-κB, TGF-β/Smad, mitochondrial glutathione and HSP responses	Hepatic fibrosis models in rats and mice; HSC and macrophage/Kupffer-like cell studies	Preclinical (*in-vitro* and animal studies)	Enhances antioxidant defense, suppresses inflammatory signaling and inhibits HSC activation

Future work should focus on standardized experimental systems, rigorous mechanistic validation, pharmacokinetic characterization, and clinically meaningful endpoints. In particular, studies should distinguish between prevention of fibrotic progression and true reversal of established fibrosis. In addition, combination strategies, tissue-targeted delivery, and patient stratification may improve translational relevance. A more cautious and evidence-based approach will be essential before these compounds can be considered for broader clinical application.

## 6 Limitations

Most evidence supporting these metabolites comes from *in-vitro* and animal studies, which do not fully reproduce human liver fibrosis. Study designs, doses, models, and treatment durations vary widely, limiting direct comparison across reports. For several compounds, conclusions are based mainly on changes in signaling markers rather than on definitive mechanistic or histological proof of fibrosis reversal. Clinical data remain limited, and some findings derive from complex formulations rather than isolated metabolites, making attribution difficult. In addition, fibrosis stage strongly affects reversibility; advanced fibrosis and cirrhosis involve extensive matrix crosslinking and persistent stellate cell activation, which reduce the likelihood of complete regression. Future studies should therefore use standardized models, stage-specific endpoints, and well-designed clinical trials.
